# Stress T1 mapping and quantitative perfusion cardiovascular magnetic resonance in patients with suspected obstructive coronary artery disease

**DOI:** 10.1093/ehjci/jeaf059

**Published:** 2025-02-17

**Authors:** S Borodzicz-Jazdzyk, G W de Mooij, C E M Vink, M A van de Wiel, M Benovoy, M J W Götte

**Affiliations:** Department of Cardiology, Amsterdam UMC, Vrije Universiteit Amsterdam, Amsterdam Cardiovascular Sciences, De Boelelaan 1118, 1081 HV Amsterdam, The Netherlands; 1st Department of Cardiology, Medical University of Warsaw, Banacha 1a Str., 02-097 Warsaw, Poland; Department of Cardiology, Amsterdam UMC, Vrije Universiteit Amsterdam, Amsterdam Cardiovascular Sciences, De Boelelaan 1118, 1081 HV Amsterdam, The Netherlands; Department of Cardiology, Amsterdam UMC, Vrije Universiteit Amsterdam, Amsterdam Cardiovascular Sciences, De Boelelaan 1118, 1081 HV Amsterdam, The Netherlands; Department of Epidemiology and Data Science, Amsterdam Public Health Research Institute, Amsterdam University Medical Centers, Amsterdam, The Netherlands; Area19 Medical Inc., Montreal H2V2X5, Canada; Department of Cardiology, Amsterdam UMC, Vrije Universiteit Amsterdam, Amsterdam Cardiovascular Sciences, De Boelelaan 1118, 1081 HV Amsterdam, The Netherlands

**Keywords:** cardiovascular magnetic resonance, T1 mapping, quantitative perfusion, myocardial ischaemia, coronary artery disease

## Abstract

**Aims:**

T1 mapping reactivity (ΔT1) has been proposed as a novel contrast-free technique to detect obstructive coronary artery disease (CAD). The aims of the study are: (i) to compare the cardiovascular magnetic resonance (CMR)-derived ΔT1 with quantitative perfusion (QP CMR) measures; (ii) to assess the influence of sex and comorbidities on ΔT1; and (iii) to assess the diagnostic accuracy of ΔT1 to detect obstructive CAD diagnosed with the invasive coronary angiography (ICA) and/or fractional flow reserve.

**Methods and results:**

This study retrospectively analysed 51 patients with suspected obstructive CAD who underwent CMR including rest and adenosine stress first-pass perfusion and native T1 mapping (MOLLI). A moderate correlation was found between pooled rest and stress native T1 mapping and myocardial blood flow (Pearson’s *r* = 0.476; *P* < 0.001). When stratified by myocardial perfusion reserve (MPR), ischaemic myocardium had significantly lower stress T1 mapping values (*P* < 0.001) and ΔT1 (*P* = 0.005) vs. nonischaemic myocardium. Male sex and history of diabetes were independently associated with lower ΔT1. The optimal cut-off value of ΔT1 to detect impaired MPR on a per-vessel basis was ≤5.4%, with an area under the curve of 0.662 (95% CI: 0.563–0.752, *P* = 0.003), sensitivity of 84% (95% CI: 67–95), and specificity of 46% (95% CI: 34–58). When validated against ICA, stress T1 and ΔT1 did not reach statistical significance in detecting obstructive CAD.

**Conclusion:**

ΔT1 is significantly influenced by sex and comorbidities and has poor diagnostic accuracy for detecting myocardial ischaemia. Therefore, the clinical utility of ΔT1 in a real-world cohort of patients to detect obstructive CAD is limited.

## Graphical Abstract

**Graphical Abstract:**
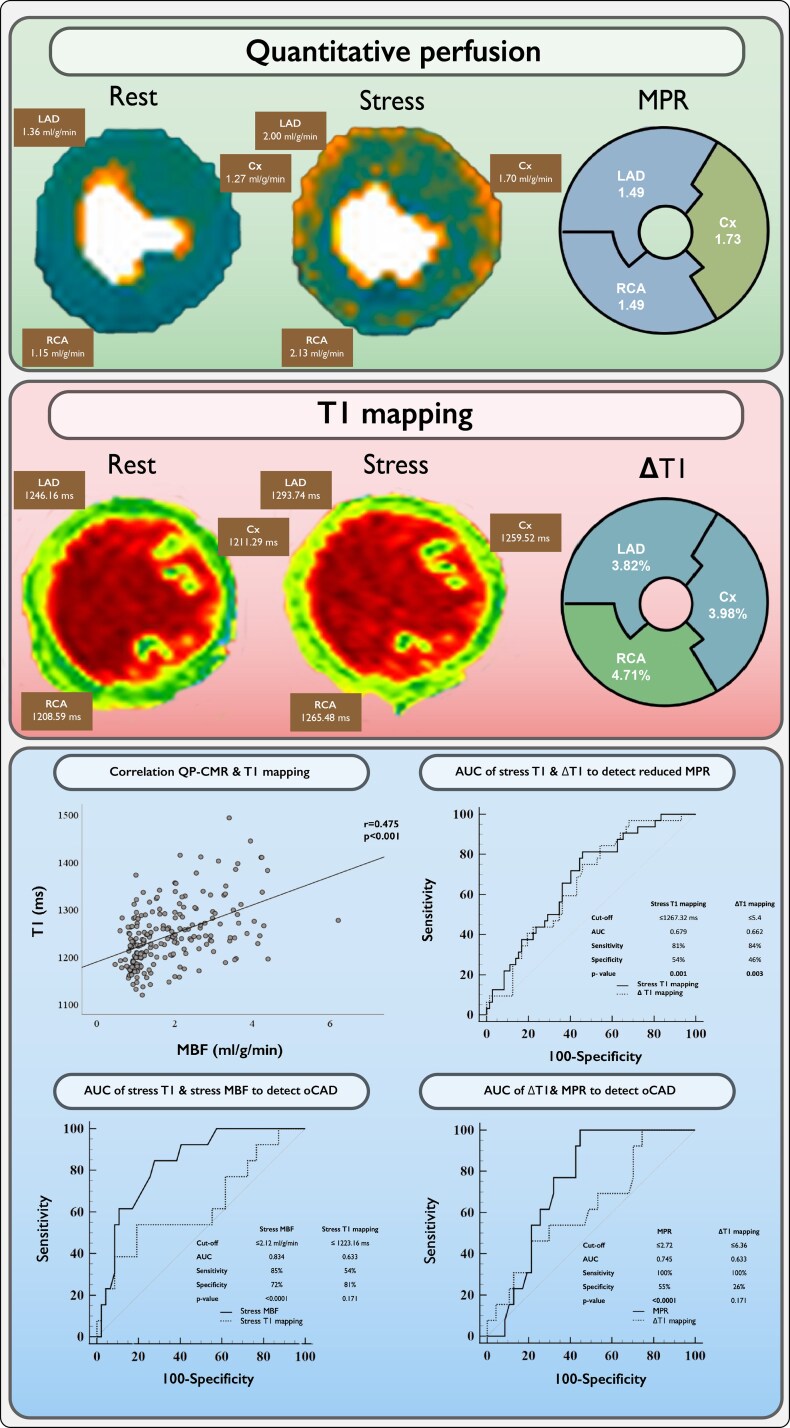
This study compared CMR-derived ΔT1 and QP measures, evaluated the influence of sex and comorbidities on ΔT1, and assessed the diagnostic accuracy of ΔT1 to detect oCAD, as diagnosed by ICA and/or fractional flow reserve, in a real-world cohort of patients with suspected oCAD. Pooled rest and stress native T1 mapping correlated moderately with MBF (Pearson’s *r* = 0.476; *P* < 0.001). The optimal cut-off value of ΔT1 to detect impaired MPR on a per-vessel basis was ≤5.4%, with an AUC of 0.662 (95% CI: 0.563–0.752, *P* = 0.003), sensitivity of 84% (95% CI: 67–95), and specificity of 46% (95% CI: 34–58). When validated against ICA, stress T1 and ΔT1 did not reach statistical significance for detecting oCAD. In contrast, stress MBF and MPR demonstrated good diagnostic accuracy for detecting oCAD with AUCs of 0.834 and 0.745, respectively. In the presented case example, myocardial ischaemia with reduced stress MBF and MPR in all coronary territories was associated with reduced ΔT1 values in the respective territories. ΔT1, T1 mapping reactivity; AUC, area under the curve; CI, confidence interval; CMR, cardiovascular magnetic resonance; ICA, invasive coronary angiography; MBF, myocardial blood flow; MPR, myocardial perfusion reserve; oCAD, obstructive coronary artery disease; QP, quantitative perfusion.

**See the editorial comment for this article ‘T1 mapping is not ready to replace the use of contrast agents in stress CMR’, by G.D. Aquaro and C. De Gori, https://doi.org/10.1093/ehjci/jeaf098.**

## Introduction

Cardiovascular magnetic resonance (CMR)-derived T1 mapping is an established method of contrast-free myocardial tissue characterization in various cardiac diseases.^1,2^ T1 mapping allows voxel-based measurement of the longitudinal time constant (T1 relaxation), which is highly dependent on the composition of surrounding tissue. Myocardial native T1 mapping represents a composed signal from the intracellular (myocytes) and extracellular space (interstitial and intravascular spaces).^3^ Large amounts of free water within the cardiac muscle prolong the T1 relaxation, while iron or lipid myocardial deposition shortens it.^1,4^ Since myocardial water content may increase due to coronary vasodilation, native T1 mapping has been proposed as a biomarker to reflect the cardiac microvascular state and myocardial blood volume (MBV), which represents the total amount of blood in the arterioles, capillaries, and venules.^3,5,6^

ΔT1 has been proposed as a novel contrast-free technique to detect obstructive coronary artery disease (CAD).^7–9^ From the pathophysiological point of view, haemodynamically significant coronary artery stenosis leads to capillary recruitment and compensatory downstream vasodilation, which results in a stationary increased MBV and free water capacity within myocardium. In such scenario, resting native T1 values are expected to be elevated and not show further substantial increase during stress-induced vasodilation what results in blunted ΔT1 when compared with healthy myocardium.^3,5^

Although experimental studies have shown excellent ability of ΔT1 to detect myocardial ischaemia, clinical studies have shown conflicting results, making its true clinical utility uncertain.^10–12^ Moreover, the influence of sex and comorbidities on both MBV and myocardial perfusion in patients with suspected obstructive CAD remains unclear. Finally, only very limited data on validation of ΔT1 against invasive coronary angiography (ICA) for detecting obstructive CAD is available.^7^ Therefore, the aims of the current study were: (i) to compare the CMR-derived ΔT1 with quantitative perfusion (QP CMR) measures; (ii) to assess the influence of sex and comorbidities on ΔT1; and (iii) to assess the diagnostic accuracy of ΔT1 to detect obstructive CAD diagnosed with the golden standard ICA and/or fractional flow reserve (FFR).

## Methods

### Study population

This study included patients with suspected obstructive CAD referred to the Amsterdam University Medical Centers, location VUmc between November 2021 and 2023 for adenosine stress perfusion CMR. Data on ICA and/or FFR performed as part of clinical management were collected where available. Patients were eligible for inclusion if both stress and rest native T1 mapping were acquired during the CMR exam, and a dual-bolus contrast administration scheme was used. Patients with a history of coronary artery bypass grafting surgery were excluded from the analysis. The study complies with the 1964 Helsinki Declaration and its later amendments and was approved by the Medical Ethics Review Committee of the Amsterdam UMC, location VUmc. All patients provided written informed consent.

### CMR image acquisition

**Figure 1:**
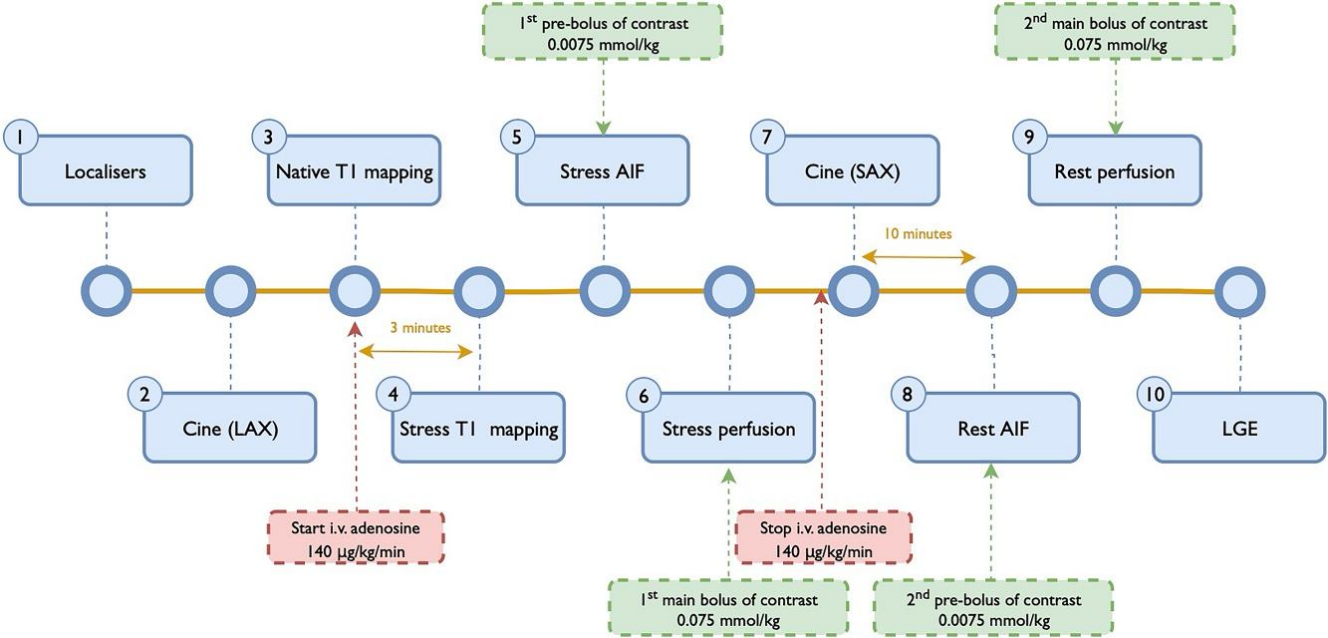
Adenosine stress perfusion CMR scanning protocol. AIF, arterial input function; LAX, long axis; LGE, late gadolinium enhancement; SAX, short axis.

Patients were instructed to avoid consuming products containing caffeine or xanthine for a minimum 24 h before the examination. Images were obtained on a 3 T whole-body MR scanner (Magnetom Vida, Siemens, Healthcare, Erlangen, Germany). CMR protocol is presented in *Figure 1*. Left ventricular (LV) systolic function was assessed using the balanced steady-state free precession cine imaging in the two-, three-, and four-chamber long-axis and short-axis stack images covering the LV from the base to the apex. Native T1 maps were acquired using the 5(3)3 MOLLI sequence at rest (baseline) and after at least 3 min of constant intravenous infusion of adenosine at a dose of 140 μg/kg/min in three parallel short-axis slices at the basal, mid-ventricular, and apical levels in diastole. Typical in-plane resolution of T1 mapping images was 1.4 × 1.4 mm^2^ with a slice thickness 8 mm (time of echo 1.06 ms, time of repetition 2.54 ms, time of inversion 180 ms, matrix size 256 × 169, field of view 320–400 mm, flip angle 35°) and a two-fold acceleration using GRAPPA/T-pat. Raw MOLLI images and maps were inspected after acquisition and T1 mapping images were repeated if the quality was assessed insufficient.

Stress and rest perfusion images were acquired in corresponding diastolic phase, according to the dual-bolus GBCA administration scheme as previously described in detail.^13^ Late gadolinium enhancement (LGE) images were acquired 10–15 min after rest perfusion using a 2D segmented inversion-recovery gradient-echo pulse sequence.

### Invasive coronary angiography and fractional flow reserve measurements

ICA was performed using a standard protocol in at least two orthogonal directions per evaluated coronary artery segment. To induce epicardial coronary vasodilation, 0.2 mL of intracoronary nitroglycerine was injected before contrast administration. FFR measurements during ICA were performed at the discretion of the operator, and according to the currently available guidelines and recommendations. To induce maximal coronary hyperaemia, intracoronary (150 µg) adenosine infusion was used. FFR was calculated as the ratio of the mean distal intracoronary pressure, to the mean arterial pressure measured by the coronary catheter.

Obstructive CAD was defined as a FFR ≤ 0.80, or ≥70% diameter stenosis (DS) based on visual assessment of ICA if FFR measurements were not performed. Left main lesions were assumed being obstructive at ≥50% DS in the absence of FFR. Accordingly, a non-obstructive coronary artery was defined as an FFR > 0.80 or DS < 70%.

### CMR image post-processing and analysis

Data on LV volumes, function, and the LGE assessment were part of a clinical analysis, supervised by level 3 expert physicians with >10 years of experience in CMR. For the QP and T1 map analysis, each scan was anonymized and analysed by one experienced level 3 CMR reader (S.B.-J.), who was blinded to the clinical data and the results of other imaging techniques and results. Post-processing of CMR images (including T1 mapping and QP) was performed using the cvi42 software (Circle Cardiovascular Imaging Inc., Calgary, Canada).

Initially, T1 map raw MOLLI images were used for quality control before post-processing, including the assessment of off-resonance and movements artefacts. Endocardial and epicardial contours were placed automatically by the software with a 10% offset on endo- and epicardial borders and corrected manually by the operator with care taken to avoid partial volume effects from the blood pool and other adjacent tissues. Rest and stress T1 maps were divided to automatically determined myocardial segments according to the American Heart Association’s 16-segment model. Myocardial segments deemed non-diagnostic due to presence of artefacts were excluded from the analysis. ΔT1 was calculated as:


$$\Delta T1\;=\frac{\left(\mathrm{stress}\;T1-\mathrm{rest}\;T1\right)}{\mathrm{rest}\;T1}\;\times 100\text{\%}.$$

Post-processing of QP CMR was performed using the pixel-wise QP module.^14–16^ Individual components of the software were described in detail elsewhere.^14,17–20^ Briefly, the correction of motion and coil-induced signal inhomogeneities was performed automatically by the software.^17,18^ Subsequently, arterial input function and myocardial regions of interest (ROIs) were detected automatically to delineate the time-signal intensity curves.^19^ Furthermore, the software performed the model-constrained deconvolution process on a pixel-by-pixel basis to assess myocardial blood flow (MBF) and provide quantitative MBF pixel maps.^14,20^ The operator (S.B.-J.) was allowed to manually correct inaccurate automatic delineation of myocardial ROIs and care was taken to exclude the blood pool areas from the ROIs. QP CMR measures included stress and rest MBF (mL/g/min) and myocardial perfusion reserve (MPR; calculated as a ratio of stress MBF over rest MBF). Similarly to T1 mapping, these parameters were calculated for automatically determined myocardial segments according to the American Heart Association’s 16-segment model. Adequate response to adenosine was assessed based on stress MBF in QP, heart rate during adenosine-induced stress, and presence of the splenic switch-off phenomenon. The previously established cut-off value of ≤1.94 mL/g/min for stress MBF and ≤1.96 for MPR was used to define myocardial ischaemia on a per-coronary territory basis.^21^ Additional per-vessel analyses were performed using the vessel-specific cut-off values, for LAD: MBF ≤ 1.88 mL/g/min, MPR ≤ 1.54; for RCA: MBF ≤ 1.50 mL/g/min, MPR ≤ 2.15; and for Cx: MBF ≤ 2.01 mL/g/min, MPR ≤ 2.15.^21^ Coronary territories with presence of LGE were excluded from the analysis.

### Statistical analysis

Statistical analysis was performed with SPSS software package (IBM SPSS Statistics 20.0, Chicago, IL, USA) and MedCalc (MedCalc Software 12.7.8.0, Mariakerke, Belgium). Continuous variables are expressed as mean ± standard deviation (SD). QQ-plots did not indicate strong deviations from the normal distribution for all outcomes. Paired samples *t*-tests were used to compare T1 mapping and QP parameters at rest and stress at patient level, including all coronary territories in the analysis (also those with the presence of LGE). For the per-vessel analyses, coronary territories with LGE were excluded, and a standardized approach was applied across all patients without accounting for individual anatomical variability in coronary vascularization (such as variations in right or left coronary dominance). T1 mapping and QP measures in different coronary artery territories were compared using two-way analysis of variance (ANOVA) with patient as a blocking variable. Correlation between T1 mapping and MBF was performed using Pearson’s correlation that effectively quantifies the sum of the within-participant and across-participant correlation. Comparison of T1 mapping and QP measures between ischaemic and nonischaemic coronary territories was performed with independent samples *t*-test. Linear mixed model analyses were performed where the dependent variables were rest and stress T1 mapping, MBF, MPR, and ΔT1; the independent variables were sex, history of hypertension, history of diabetes, history of smoking, with age and left ventricular mass as covariates, and patient as a subject grouping variable. A receiver operating characteristics (ROC) curve per-vessel analysis was performed to assess the diagnostic accuracy of T1 mapping to detect myocardial ischaemia (defined as hypoperfusion in QP or presence of significant coronary artery obstruction in ICA). The Youden index was used to identify the optimal T1 mapping cut-off values on a per-vessel basis using the mean rest, stress T1 mapping, or ΔT1 value of a coronary territory. Per-vessel diagnostic performance measures [sensitivity, specificity, negative predictive value (NPV), positive predictive value (PPV)] were calculated including 95% confidence interval. All statistical tests were two-tailed, and a *P*-value of <0.05 was considered significant.

## Results

### Study population

In total, 56 patients met the eligibility criteria. Three cases were excluded due to insufficient response to adenosine and two cases were excluded because of technical issues with the QP post-processing. Consequently, the final analysis comprised 51 patients. Baseline characteristics of the study population are summarized in *Table 1*, and CMR parameters of left and right ventricular dimensions and functions are presented in Supplementary data online, *Table S1*. The mean age was 61 ± 10 years, with 57% of the patients being male. ICA was performed in 31 patients (61%), and eight (26%) of them had FFR measurements performed. In six patients, rest and stress T1 mapping were acquired solely at the mid-ventricular level, in one patient, due to technical problems, rest mapping was acquired at mid-ventricular level while stress at basal level, and in one patient, T1 mapping was acquired at the basal and mid-ventricular levels. Due to presence of artefacts, 22 segments (3.0%) in rest T1 mapping and 72 segments (9.7%) in stress T1 mapping were deemed of insufficient quality and excluded from the analysis. Sixteen patients were diagnosed with obstructive CAD on ICA (52%). For the per-vessel analysis, 48 coronary territories were excluded due to the presence of LGE, leaving a total of 105 coronary territories for analysis. Of these, 10 (10%) were assessed with FFR, and 13 (12%) were deemed to have a significant stenosis.

**Table 1:** Baseline characteristics of the patient cohort

Number of patients	51
Demographic data	
Age (years)	61 ± 10
Male gender	29 (57%)
BMI (kg/m^2^)	25.8 ± 4.0
Medical history and risk factors	
Known CAD history	23 (45%)
Prior PCI	16 (31%)
Prior MI	13 (26%)
Hypertension	22 (43%)
Dyslipidaemia	15 (29%)
Diabetes mellitus	8 (16%)
Smoking	18/50 (36%)
Family history of CAD	14/49 (29%)
Medication	
Aspirin	29/50 (58%)
Beta-blockers	21/50 (42%)
ACEI or ARB	18/50 (36%)
Calcium channel blockers	17/50 (34%)
Statin	28/50 (56%)

Categorical variables are presented as number and percentage. Continuous variables are expressed as mean ± SD.

ACEI, angiotensin converting enzyme inhibitors; ARB, angiotensin receptor blockers; BMI, body mass index; CAD, coronary artery disease; MI, myocardial infarction; PCI, percutaneous coronary intervention.

### Correlation between T1 mapping and MBF

At rest, global (per-patient) mean native T1 mapping was 1222 ± 49 ms and after administration of adenosine increased to 1279 ± 63 ms (*P* < 0.001), resulting in ΔT1 of 4.67 ± 3.23%. A per-vessel subanalysis of different coronary artery territories revealed significant differences in rest and stress T1 mapping, while ΔT1 did not differ between the three main coronary artery territories (see Supplementary data online, *Table S2*). Global (per-patient) mean rest MBF was 1.09 ± 0.26 mL/g/min which at stress increased to 2.51 ± 0.85 mL/g/min (*P* < 0.001), yielding an MPR of 2.47 ± 0.94. In a per-vessel analysis, among other coronary territories, the LAD had the highest rest and stress MBF and MPR (see Supplementary data online, *Table S2*).

**Figure 2:**
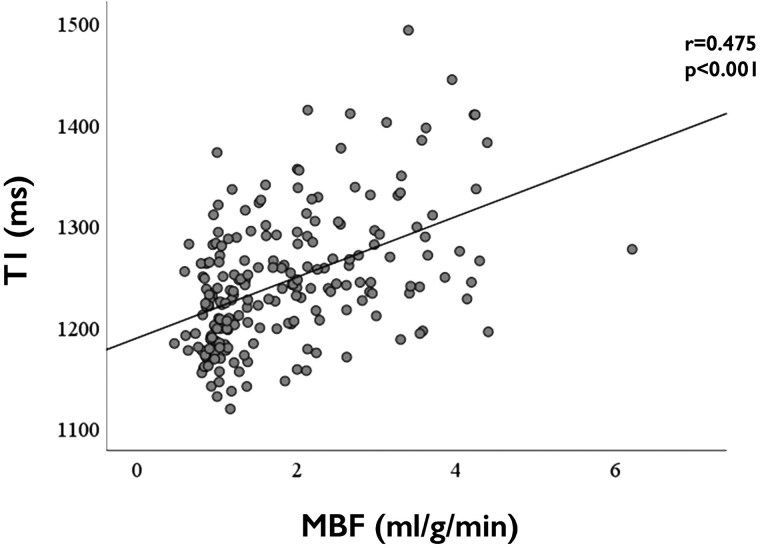
Correlation between T1 mapping and quantitative perfusion measures. MBF, myocardial blood flow.

A significant, but moderate correlation was found between pooled rest and stress native T1 mapping and MBF (Pearson’s *r* = 0.475; *P* < 0.001, *Figure 2*). Moreover, significant correlations were found between rest and stress T1 mapping (Pearson’s *r* = 0.759, *P* < 0.001), stress T1 and stress MBF (Pearson’s *r* = 0.269, *P* = 0.005), stress T1 and ΔT1 (Pearson’s *r* = 0.607, *P* < 0.001), rest MBF and MPR (Pearson’s *r* = −0.478, *P* < 0.001), stress MBF and MPR (Pearson’s *r* = 0.797, *P* < 0.001), stress MBF and ΔT1 (Pearson’s *r* = 0.299, *P* = 0.002), and MPR and ΔT1 (Pearson’s *r* = 0.321, *P* < 0.001) (see Supplementary data online, *Figure S1*).

### T1 mapping in ischaemic myocardium

**Figure 3:**
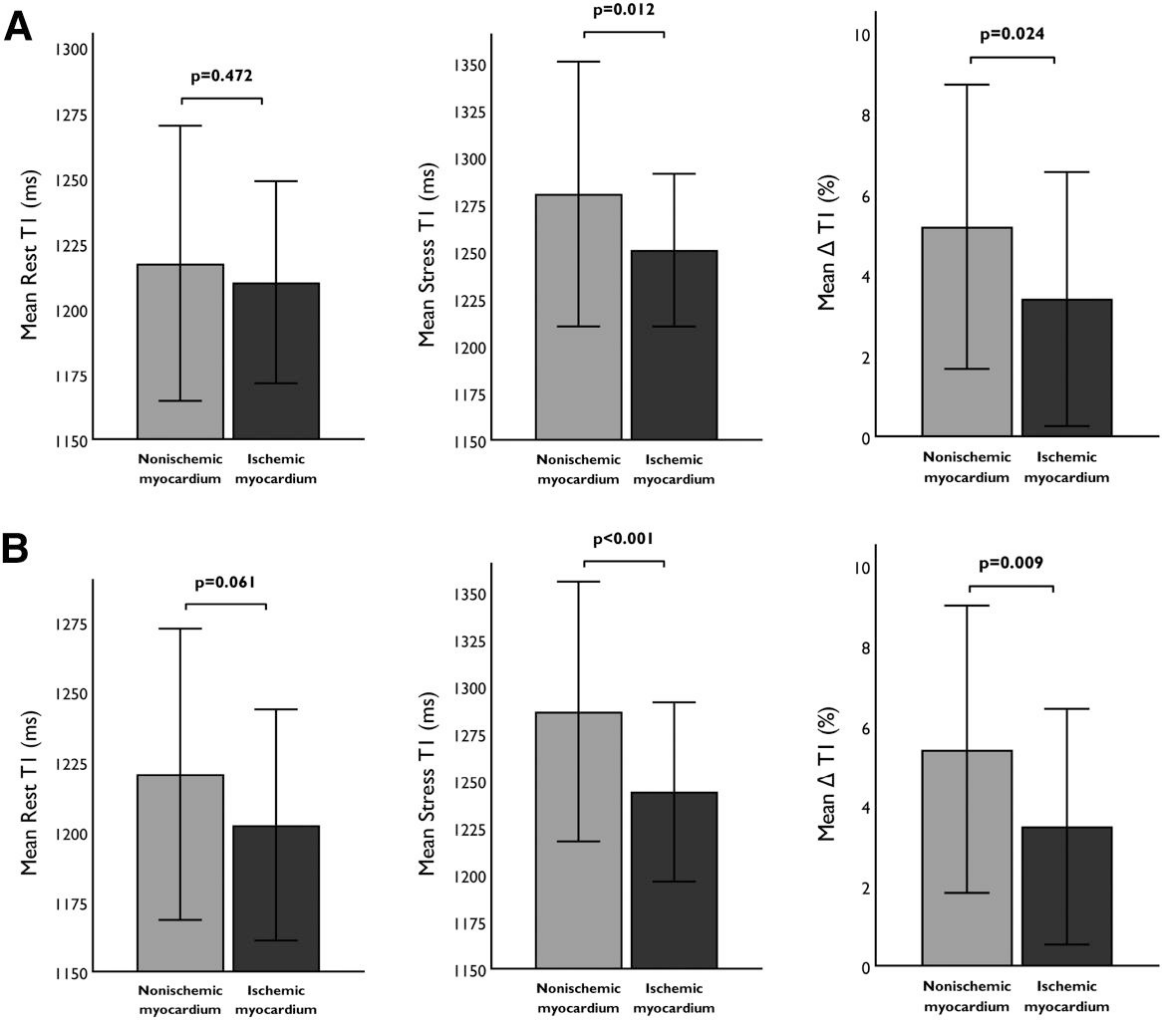
Native T1 mapping in ischaemic myocardium. Panel *A* shows rest and stress T1 mapping and ΔT1 in ischaemic and nonischaemic myocardium when stratified by MBF, while panel *B* shows stratification according to MPR. MBF, myocardial blood flow; MPR, myocardial perfusion reserve; ΔT1, T1 mapping reactivity.

When stratified by MBF, ischaemic and nonischaemic coronary territories did not differ in rest T1 mapping (1210.13 ± 38.74 vs. 1217.34 ± 52.69 ms, *P* = 0.472). However, ischaemic myocardium had significantly lower stress T1 mapping values (1250.71 ± 40.38 vs. 1280.47 ± 70.23 ms, *P* = 0.012) and ΔT1 (3.40 ± 3.14 vs. 5.19 ± 3.52%, *P* = 0.024) when compared with nonischaemic myocardium (*Figure 3*). Similarly, when stratified according to MPR, ischaemic and nonischaemic myocardium did not differ in rest T1 mapping (1202.45 ± 41.28 vs. 1220.60 ± 52.07, *P* = 0.061). Ischaemic coronary territories had significantly lower stress T1 mapping values (1243.90 ± 47.63 vs. 1286.59 ± 68.99 ms, *P* < 0.001) and ΔT1 (3.48 ± 2.95 vs. 5.41 ± 3.59, *P* = 0.009; *Figure 3*).

Stratification according to ICA revealed lower mean rest, stress T1, and ΔT1 values in myocardium supplied by a coronary artery with a significant stenosis when compared with non-obstructive coronary artery territory, however these observations did not reach statistical significance (rest T1: 1194.65 vs. 1200.67 ms, *P* = 0.620; stress T1: 1230 vs. 1257.76 ms, *P* = 0.078; ΔT1: 2.99 vs. 4.78, *P* = 0.096).

### Influence of sex and comorbidities on T1 mapping and myocardial perfusion

Male sex was independently associated with lower stress T1 (1246.41 ± 51.89 ms vs. 1301.48 ± 67.73 ms, *P* = 0.006) and ΔT1 (3.75 ± 3.48 vs. 5.90 ± 3.23, *P* = 0.01). In addition, diabetes was an independent predictor of lower ΔT1 (2.75 ± 3.62 vs. 5.21 ± 3.37, *P* = 0.047). Presence of hypertension was significantly associated with lower stress MBF (2.17 ± 0.67 vs. 2.83 ± 0.97, *P* = 0.039) and lower MPR (2.14 ± 1.11 vs. 2.81 ± 0.84, *P* = 0.027; *Table 2*).

**Table 2:** Results from linear mixed model analyses for myocardial T1 mapping and quantitative perfusion

Model^a^	Stress T1				ΔT1				Stress MBF				MPR			
	Estimate^b^	df	*P*-value	95% CI	Estimate^b^	df	*P*-value	95% CI	Estimate^b^	df	*P*-value	95% CI	Estimate^b^	df	*P*-value	95% CI
Intercept	1045.27	36.43	<0.001	797.48–1293.06	−10.17	33.76	0.130	−23.50–3.15	0.99	34.62	0.577	−2.60–4.59	0.95	35.48	0.643	−3.19–5.09
Sex	79.67	36.16	0.006	24.53–134.81	3.97	33.35	0.010	1.01–6.93	0.14	34.23	0.726	−0.66–0.94	−0.06	35.36	0.890	−0.98–0.86
Hypertension	23.21	37.82	0.295	−21.01–67.44	0.60	36.17	0.616	−1.80–3.00	0.68	36.87	0.039	0.04–1.33	0.84	36.11	0.027	0.10–1.58
Diabetes	13.07	37.04	0.594	−36.23–62.36	2.70	35.05	0.047	0.04–5.36	0.35	35.78	0.331	−0.37–1.06	0.18	35.80	0.658	−0.64–1.00
Current or past smoking	35.24	37.87	0.090	−5.81–76.29	1.52	36.34	0.174	−0.70–3.75	0.52	37.02	0.084	−0.07–1.12	0.46	36.24	0.183	−0.23–1.14
LV mass	0.91	36.74	0.061	−0.04–1.86	0.05	34.30	0.067	0–0.10	0.00	35.12	0.556	−0.02–0.01	0.00	35.63	0.672	−0.02–0.01
Age	0.82	36.68	0.463	−1.42–3.05	0.08	34.17	0.208	−0.04–0.20	0.02	35.01	0.347	−0.02–0.05	0.02	35.63	0.367	−0.02–0.05

ΔT1, T1 mapping reactivity; MBF, myocardial blood flow; MI, myocardial infarction; MPR, myocardial perfusion reserve.

^a^The model included: sex, hypertension, diabetes, current or past smoking, and LV mass and age as covariates.

^b^Estimates comparisons for the effects are given as: male vs. female; hypertension vs. no hypertension; diabetes vs. no diabetes; current or past smoking vs. no current or past smoking.

### Diagnostic performance of T1 mapping to detect myocardial ischaemia

**Figure 4:**
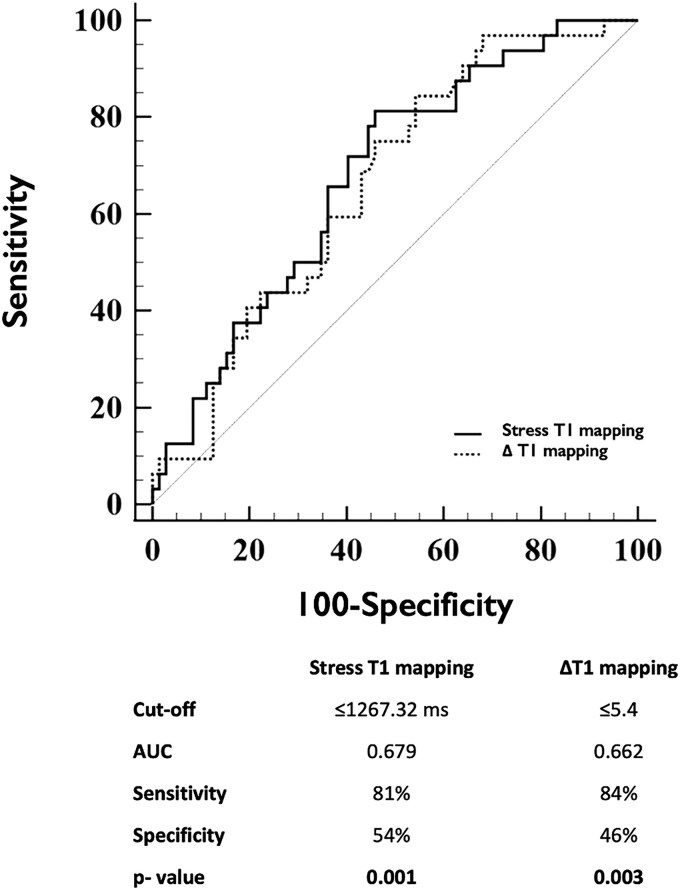
Diagnostic performance of stress T1 mapping and ΔT1 to detect myocardial ischaemia (defined as reduced MPR) on a per-coronary territory analysis. AUC, area under the curve; MPR, myocardial perfusion reserve; ΔT1, T1 mapping reactivity.

**Figure 5:**
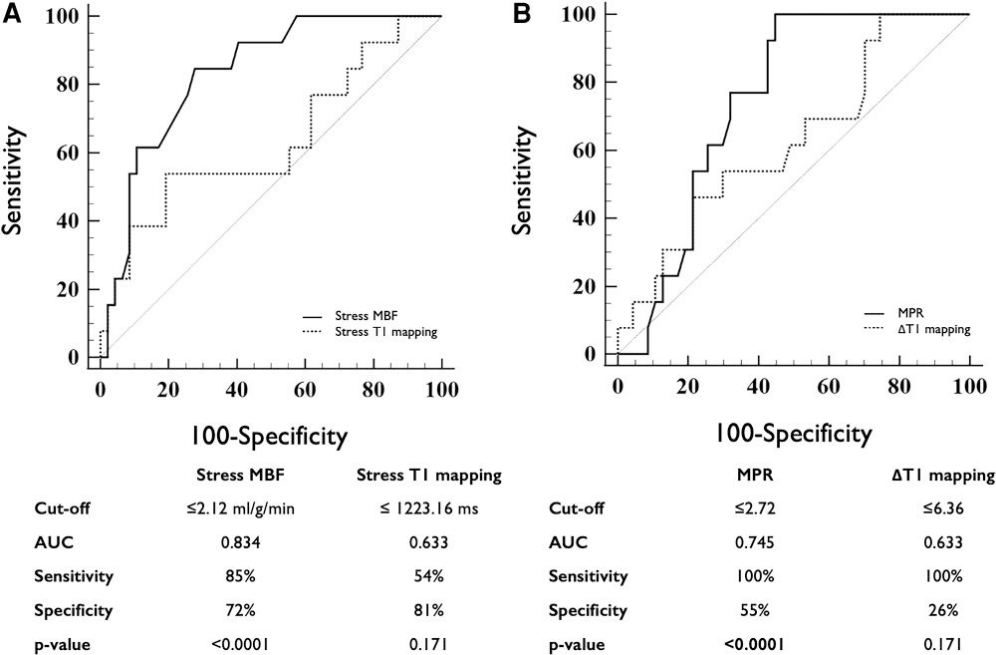
Diagnostic performance of T1 mapping and quantitative perfusion measures to detect obstructed coronary artery on a per-coronary territory analysis. AUC, area under the curve; MBF, myocardial blood flow; MPR, myocardial perfusion reserve; ΔT1, T1 mapping reactivity.

Diagnostic performance and ROC curves of T1 mapping measures to detect impaired myocardial perfusion on a per-coronary territory basis are shown in *Table 3*, Supplementary data online, *Table S3* and *Figure 4*. When all vessels were analysed, stress T1 mapping and ΔT1 were able to identify impaired MPR, but not MBF. The optimal cut-off value of stress T1 to detect impaired MPR on a per-vessel basis was ≤1267.32 ms, with a corresponding area under the curve (AUC) of 0.679 (95% CI: 0.580–0.767, *P* = 0.001), sensitivity of 81% (95% CI: 64–93), specificity of 54% (95% CI: 42–66), PPV 44% (95% CI: 37–52%), and NPV 87% (95% CI: 75–93%) (*Figure 4*). The optimal cut-off value of ΔT1 to detect impaired MPR on a per-vessel basis was ≤5.4%, with a corresponding AUC of 0.662 (95% CI: 0.563–0.752, *P* = 0.003), sensitivity of 84% (95% CI: 67–95), specificity of 46% (95% CI: 34–58), PPV 41% (95% CI: 35–47%), and NPV 87% (95% CI: 74–94%) (*Figure 4*). When validated against ICA, stress T1 and ΔT1 failed to reach the statistical significance to detect obstructive CAD (*Table 4* and *Figure 5*).

**Table 3:** Diagnostic accuracy of T1 mapping parameters to detect myocardial ischaemia defined as reduced MPR

	*n*	AUC (95% CI)	Optimal cut-off	Sensitivity (%)	Specificity (%)	*P*-value
ΔT1						
LAD (MPR ≤ 1.54)	36	0.772 (0.602–0.895)	≤3.82	83 (36–100)	77 (58–90)	0.007
RCA (MPR ≤ 2.15)	34	0.634 (0.452–0.792)	≤6.85	100 (74–100)	32 (14–55)	0.185
Cx (MPR ≤ 2.15)	35	0.605 (0.426–0.766)	≤5.39	82 (54–96)	47 (24–71)	0.287
All vessels (MPR ≤ 1.96)	105	0.662 (0.563–0.752)	≤5.4	84 (67–95)	46 (34–58)	0.003
Stress T1						
LAD (MPR ≤ 1.54)	36	0.600 (0.424–0.759)	≤1267.32 ms	83 (36–100)	57 (37–75)	0.2981
RCA (MPR ≤ 2.15)	34	0.702 (0.540–0.860)	≤1253.35 ms	67 (35–90)	82 (60–95)	0.024
Cx (MPR ≤ 2.15)	35	0.559 (0.382–0.726)	≤1259.52 ms	75 (48–93)	47 (24–71)	0.558
All vessels (MPR ≤ 1.96)	105	0.679 (0.580–0.767)	≤1267.32 ms	81 (64–93)	54 (42–66)	0.001
Rest T1						
LAD (MPR ≤ 1.54)	36	0.544 (0.370–0.711)	>1191.45 ms	83 (36–100)	43 (26–63)	0.723
RCA (MPR ≤ 2.15)	34	0.561 (0.381–0.730)	≤1249.58 ms	83 (52–98)	36 (17–59)	0.559
Cx (MPR ≤ 2.15)	35	0.513 (0.339–0.685)	≤1155.7 ms	19 (4–46)	100 (82–100)	0.898
All vessels (MPR ≤ 1.96)	105	0.573 (0.472–0.669)	≤1254.73 ms	94 (79–99)	25 (16–37)	0.220

ΔT1, T1 mapping reactivity; AUC, area under the curve; CI, confidence interval; Cx, circumflex artery; LAD, left anterior descending artery; MPR, myocardial perfusion reserve; RCA, right coronary artery.

**Table 4:** Diagnostic accuracy of T1 mapping and quantitative perfusion measures to detect obstructive coronary artery disease

	*n*	AUC (95% CI)	Optimal cut-off	Sensitivity (%)	Specificity (%)	*P*-value
T1 mapping						
ΔT1	60	0.627 (0.492–0.748)	≤6.36	100 (75–100)	26 (14–40)	0.150
Stress T1	60	0.633 (0.499–0.754)	≤1223.16 ms	54 (25–81)	81 (67–91)	0.171
Rest T1	60	0.545 (0.411–0.674)	≤1141.22 ms	23 (5–54)	98 (89–100)	0.654
Quantitative perfusion						
MPR	60	0.745 (0.617–0.849)	≤2.72	100 (75–100)	55 (40–70)	0.0001
Stress MBF	60	0.834 (0.715–0.917)	≤2.12 mL/g/min	85 (55–98)	72 (57–84)	<0.0001
Rest MBF	60	0.529 (0.395–0.659)	≤1.18 mL/g/min	85 (55–98)	30 (17–45)	0.746

ΔT1, T1 mapping reactivity; AUC, area under the curve; CI, confidence interval; MBF, myocardial blood flow; MPR, myocardial perfusion reserve.

## Discussion

This study assessed the agreement between the CMR-derived T1 mapping with QP CMR measures, assessed the influence of sex and comorbidities on T1 mapping, and evaluated the diagnostic accuracy of T1 mapping for detecting myocardial ischaemia. In the study cohort, there was only moderate correlation between T1 mapping and QP measures. In addition, the study has shown that male sex and diabetes are independently associated with lower ΔT1, what may lead to false positive diagnosis of myocardial ischaemia in the daily clinical practice of patients with suspected obstructive CAD. Finally, the study has shown that although the values of stress T1 mapping and ΔT1 are lower in ischaemic myocardium than in nonischaemic territories, these parameters have poor diagnostic accuracy for detecting myocardial ischaemia or presence of a significant coronary obstruction.

In healthy cohort, native T1 correlates strongly with MBF (*R*^2^ = 0.53) and extracellular volume (ECV; *R*^2^ = 0.57).^5^ Intravenous administration of adenosine increases values of native T1 mapping, MBF, MBV, and ECV, where the change in native T1 mapping is best explained by change in ECV (beta 0.50) and MBF (beta 0.43, model *R*^2^ = 0.69).^5^ Physiologically, increase in adenosine stress T1 mapping and MBF are mediated by different receptors and signalling pathways, therefore they are not equivalent, what should be considered in their clinical interpretation.^22^ Experimental studies demonstrated that adenosine-induced increase in MBF is mediated exclusively through A_2A_ adenosine receptor (AR) and is independent of endothelial nitric oxide synthase (eNOS), whereas ΔT1 is mediated through two types of AR—A_2A_ and A_2B_ AR, and is partially eNOS dependent through A_2A_AR-mediated pathway.^22^ It has been hypothesized that AR subtypes and eNOS differentially affect MBF and ΔT1 due to their role in different coronary artery segments. Increased MBF results from A_2A_AR-dependent vasodilation in arterioles, independent of the endothelium. In contrast, adenosine-induced increases in MBV and stress T1 involve two mechanisms: A_2A_AR-mediated, endothelium-dependent capillary recruitment and dilation, and A_2B_AR-mediated, endothelium-independent relaxation of vascular smooth muscle in terminal arterioles.^22^

Unlike in healthy cohorts, where native T1 mapping strongly correlates with MBF, the correlation in patients with suspected CAD is only moderate, as shown in both PET studies and the current QP CMR study.^5,11^ This is consistent with the pathophysiological adaptations to presence of a significant coronary artery stenosis, where resting MBF remains constant due to autoregulatory vasodilation of the microvasculature distal to the stenosis, leading to an increase in MBV.^9,23^ The increase in MBV is nonlinear with increasing degrees of coronary stenosis until the stenosis becomes critical. At moderate levels of coronary stenosis, MBV remains constant despite ongoing autoregulation, due to a reduction in the size of the perfusion bed supplied by the stenotic vessel. Once autoregulation is exhausted, a decrease in MBV is observed with further increases in coronary stenosis. Although our study showed that myocardial hypoperfusion is accompanied by reduced stress T1 mapping and blunted ΔT1, the complex adaptive mechanisms and nonlinear relation between MBV and MBF in the presence of coronary stenosis make the native T1 mapping not a reliable marker of myocardial perfusion in patients with obstructive CAD.

As MBV represents the total volume of blood in both the micro- and macrocirculation, it has been proposed as a more comprehensive marker of myocardial ischaemia than MBF alone.^7^ Experimental studies demonstrated that during dobutamine stress, MBV correlates more strongly with total myocardial oxygen demand than MBF and is a reliable marker for detecting significant coronary artery stenosis and assessing functional relevance of coronary stenoses of intermediate angiographic severity.^9,24,25^ Interestingly, experimental and clinical studies give conflicting results regarding the utility of native T1 mapping to detect myocardial ischaemia. A histopathologic validation study using miniature swine to assess stress T1 mapping for myocardial ischaemia induced by mechanically induced LAD stenosis demonstrated excellent diagnostic accuracy of ΔT1 for detecting ischaemic (AUC 0.89; 95% CI: 0.83–0.93) and infarcted (AUC 0.97; 95% CI: 0.92–0.99) myocardium.^10^ In patients with angiographically significant stenosis (>50%) in ≥1 coronary artery and healthy volunteers, adenosine stress and rest native T1 mapping have shown the ability to differentiate between ischaemic, infarcted, remote, and normal myocardium.^7^ Ischaemic myocardium had elevated rest T1 mapping values compared with the myocardium of healthy volunteers, without significant adenosine-induced ΔT1. Moreover, infarcted myocardium showed the highest rest T1, also without significant ΔT1, while remote myocardium had rest T1 values comparable to normal myocardium, but with a blunted ΔT1 response.^7^ Further clinical studies confirmed these observations, also in patients undergoing regadenoson stress perfusion CMR.^8,12,26,27^ In our study, although ischaemic (hypoperfused) myocardium had significantly lower stress T1 mapping and ΔT1 than nonischaemic segments, the overall diagnostic accuracy of this technique for detecting ischaemic myocardium was poor, similarly to other reports where the AUC to detect inducible myocardial ischaemia by regadenoson-induced ΔT1 in patients with suspected or known CAD was 0.60 (0.58–0.62).^28^ Moreover, similarly to other report, our study found no significant differences in rest, stress native T1 mapping and also ΔT1 between myocardial segments supplied by non-obstructive and obstructive coronary arteries.^29^

As adenosine-induced ΔT1 is partially endothelium-dependent, we hypothesized that a possible explanation for the poor clinical utility of native stress T1 mapping and/or ΔT1 is its sex-related variability and sensitivity to common cardiovascular risk factors known to predispose individuals to coronary microvascular dysfunction (CMD). This might indeed explain the observed discrepancy in excellent utility of ΔT1 to detect significant coronary obstruction in animals that is not observed in clinical studies. CMD occurs as a consequence of functional and/or structural alterations.^30^ Functional mechanisms may involve vasodilator abnormalities and/or microvascular spasm, with impaired vasodilation potentially due to endothelium-dependent and/or independent mechanisms.^30^ Structural alterations involve luminal narrowing of the intramural arterioles and capillaries, perivascular fibrosis, and capillary rarefaction. Importantly, conventional cardiovascular risk factors, including hypertension, diabetes mellitus, dyslipidaemia, smoking, and obesity, have been shown to induce endothelial dysfunction. Increased LV mass, hypertension, and diabetes are also related to structural abnormalities of microvasculature.^30,31^ Experimental studies in rabbit models of CMD induced by microembolization spheres or diabetes mellitus Type 2 demonstrated that ATP-induced ΔT1 was significantly lower in the CMD group when compared with sham and control animals, with ΔT1 showing a high negative correlation with collagen volume fraction and a positive correlation with microvascular density.^32,33^ Also in clinical conditions, patients with even well-controlled Type 2 diabetes mellitus, without hypertension, and obstructive CAD exhibit blunted adenosine-induced ΔT1, likely reflecting CMD.^34^ Accordingly in the presented study involving patients with suspected obstructive CAD, diabetes was an independent predictor of lower ΔT1. Our study has also shown that male sex is an independent predictor of lower ΔT1, which is consistent with findings from the CMR analysis in healthy volunteers, where male sex was an independent contributor to lower stress MBV, as well as rest and stress MBF, and ECV.^35^ Therefore, male sex and presence of cardiovascular risk factors may independently contribute to lower ΔT1 values, potentially giving a false suspicion of obstructive CAD.

While ΔT1 showed poor diagnostic accuracy for detecting ICA-confirmed obstructive CAD in the current study’s cohort, QP CMR was effective in identifying significant coronary obstruction. As discussed above, in normal physiology, adenosine-induced vasodilation and increase in MBF are independent of the endothelium, therefore would be less likely sensitive to cardiovascular risk factors inducing endothelial dysfunction than partially endothelium-dependent ΔT1. Indeed, in our study, only hypertension was independently related to lower stress MBF and MPR, most probably due to known hypertension-induced structural abnormalities of the coronary vessels. It is also crucial to note that the concept of blunted ΔT1 as a reflection of myocardial ischaemia assumes that in the presence of a significant coronary obstruction, rest T1 mapping values are increased due to microvascular vasodilation distal to stenosis. However, in cases of very severe coronary obstruction, autoregulatory reserve may be exhausted and no longer able to maintain sufficient resting blood flow. This will cause the reduction in MBV and rest native T1 values, leading to paradoxically ‘normalized’ ΔT1 despite severe coronary stenosis.

## Study limitations

The major limitation of our study is a relatively small sample size and lack of the healthy cohort group. Therefore, the results should be confirmed in larger cohort prospective studies, ideally with incorporation of invasive haemodynamic functional assessment of coronary stenosis (i.e. fractional flow reserve and/or instantaneous wave-free ratio) and microvascular function (index of microcirculatory resistance, hyperaemic microvascular resistance, and/or coronary flow reserve). Secondly, due to the limited sample size, the potential effect of pharmacotherapy on myocardial perfusion parameters cannot be fully elucidated and requires further investigation in larger-scale (preferentially experimental) studies. Thirdly, our study was conducted using only one vasodilator (adenosine) and was limited to a single centre, a single vendor, and a 3 T scanner. Moreover, although QP CMR is a promising method of MBF quantification that has shown very good diagnostic accuracy to detect obstructive CAD, it still remains a research tool and lacks large-scale validation studies. We also used the previously established (not study- or centre-specific) cut-off values of stress MBF and MPR to define myocardial ischaemia on a per-coronary territory basis. Finally, although coronary territories with presence of focal fibrosis were excluded from the analysis, our study did not include the assessment of ECV, therefore we cannot assess the influence of diffuse fibrosis on the T1 mapping measurements.

## Conclusions

This study demonstrates that ΔT1 is significantly influenced by sex and cardiovascular risk factors, which are frequent in the population of patients with suspected obstructive CAD. Unlike QP CMR, ΔT1 has poor diagnostic accuracy to detect myocardial ischaemia in this patient cohort. Therefore, the clinical utility of a gadolinium contrast-free ΔT1 to detect obstructive CAD in a real-world patient population is limited. Accordingly, contrast-based methods are more reliable and should be the method of choice in the daily clinical practice of the CMR-based diagnostics of suspected obstructive CAD.

## Supplementary Material

jeaf059_Supplementary_Data

## Data Availability

The data underlying this article are available in the article and in its online supplementary material.
