# Supplementary material for: Stress T1 mapping and quantitative perfusion cardiovascular magnetic resonance in patients with suspected obstructive coronary artery disease
Source: Eur Heart J Cardiovasc Imaging. 2025 Feb 17;26(6):980–90. doi: 10.1093/ehjci/jeaf059 (PMC12123514; doi:10.1093/ehjci/jeaf059)

**Table S1. CMR-derived parameters of left and right ventricular dimensions and functions of the patient cohort.**

| **Left ventricular end-diastolic volume (ml)** | 155±42 |
| --- | --- |
| **Left ventricular end-diastolic volume indexed (ml/m2)** | 78±19 |
| **Left ventricular end-systolic volume (ml)** | 66±26 |
| **Left ventricular end-systolic volume indexed (ml/m2)** | 33±13 |
| **Left ventricular stroke volume (ml)** | 89±22 |
| **Left ventricular stroke volume indexed (ml/m2)** | 45±10 |
| **Left ventricular ejection fraction (%)** | 59±8 |
| **Right ventricular end-diastolic volume (ml)** | 154±46 |
| **Right ventricular end-diastolic volume indexed (ml/m2)** | 77±18 |
| **Right ventricular end-systolic volume (ml)** | 66±27 |
| **Right ventricular end-systolic volume indexed (ml/m2)** | 33±12 |
| **Right ventricular stroke volume (ml)** | 88±24 |
| **Right ventricular stroke volume indexed (ml/m2)** | 44±9 |
| **Right ventricular ejection fraction (%)** | 58±8 |

Continuous variables are expressed as mean±SD for normally distributed data.

**Table S2. Native T1 mapping and myocardial perfusion parameters in LAD, RCA and Cx coronary territories.**

|  | **LAD** | **RCA** | **Cx** | **p-value** |
| --- | --- | --- | --- | --- |
| ***T1 mapping*** | | | | |
| **Rest T1 (ms)** | 1210±46 | 1227±53** | 1208±49^##^ | **0.007** |
| **Stress T1 (ms)** | 1276±66 | 1282±67 | 1263±66# | **0.006** |
| **∆ T1 (%)** | 5.38±3.57 | 4.43±3.55 | 4.61±3.44 | 0.472 |
| ***Quantitative perfusion*** | | | | |
| **Rest MBF** | 1.18±0.32 | 0.98±0.24**** | 1.07±0.26****, ^###^ | **<0.001** |
| **Stress MBF** | 3.04±1.07 | 2.37±0.75**** | 2.44±0.79**** | **<0.001** |
| **MPR** | 2.78±1.09 | 2.56±0.93*** | 2.43±0.91**** | **<0.001** |

∆ T1 – T1 mapping reactivity; Cx – circumflex artery; LAD – left anterior descending artery; MBF – myocardial blood flow; MPR – myocardial perfusion reserve; RCA – right coronary artery. *p= vs. LAD; **p=0.003 vs. LAD; ***p=0.002 vs. LAD; ****p=<0.001 vs. LAD, #p=0.009 vs. RCA, ##p=0.002 vs. RCA, ### p<0.001 vs. RCA.

**Table S3. Diagnostic accuracy of T1 mapping parameters to detect myocardial ischemia defined as reduced stress MBF.**

|  | **n** | **AUC (95% CI)** | **Optimal cut-off** | **Sensitivity (%)** | **Specificity (%)** | **p-value** |
| --- | --- | --- | --- | --- | --- | --- |
| ***∆ T1*** | | | | | | |
| LAD (MBF≤1.88 ml/g/min) | 36 | 0.539 (0.365-0.706) | ≤3.07 | 50 (7-93) | 78 (60-91) | 0.809 |
| RCA (MBF≤1.50 ml/g/min) | 34 | 0.753 (0.575-0.884) | ≤1.59 | 67 (9-99) | 87 (70-96) | 0.186 |
| Cx (MBF≤2.01 ml/g/min) | 35 | 0.721 (0.544- 0.859) | ≤5.39 | 93 (66-100) | 52 (30-74) | **0.011** |
| All-vessels  (MBF≤1.94 ml/g/min) | 105 | 0.601 (0.501-0.695) | ≤6.46 | 96 (78-100) | 28 (19-39) | 0.117 |
| ***Stress T1*** | | | | | | |
| LAD (MBF≤1.88 ml/g/min) | 36 | 0.586 (0.410-0.747) | ≤1265.59 ms | 75 (19-99) | 56 (38-74) | 0.522 |
| RCA (MBF≤1.50 ml/g/min) | 34 | 0.613 (0.431-0.774) | ≤1251.57 ms | 67 (9-99) | 71 (52-86) | 0.523 |
| Cx (MBF≤2.01 ml/g/min) | 35 | 0.612 (0.433-0.772) | ≤1258.6 ms | 79 (49-95) | 52 (30-74) | 0.258 |
| All-vessels  (MBF≤1.94 ml/g/min) | 105 | 0.609 (0.509-0.703) | ≤1265.59 ms | 78 (56 – 93) | 52 (41– 64) | 0.061 |
| ***Rest T1*** | | | | | | |
| LAD (MBF≤1.88 ml/g/min) | 36 | 0.625 (0.448-0.780) | ≤1221.52 ms | 100 (40-100) | 47 (29-65) | 0.295 |
| RCA (MBF≤1.50 ml/g/min) | 34 | 0.710 (0.529-0.852) | >1231.81 ms | 100 (29-100) | 65 (45-81) | **0.020** |
| Cx (MBF≤2.01 ml/g/min) | 35 | 0.514 (0.339-0.686) | >1180.12 ms | 79 (49-95) | 43 (22-66) | 0.8946 |
| All-vessels  (MBF≤1.94 ml/g/min) | 105 | 0.517 (0.417-0.616) | ≤1254.73 ms | 91 (72-99) | 23 (15-34) | 0.788 |

∆ T1 – T1 mapping reactivity; AUC – area under the curve; CI – confidence interval; Cx – circumflex artery; LAD – left anterior descending artery; MBF – myocardial blood flow; RCA – right coronary artery.

**Figure S1. Correlation between T1 mapping and quantitative perfusion measures.** MBF – myocardial blood flow; MPR – myocardial perfusion reserve; ∆ T1 – T1 mapping reactivity.


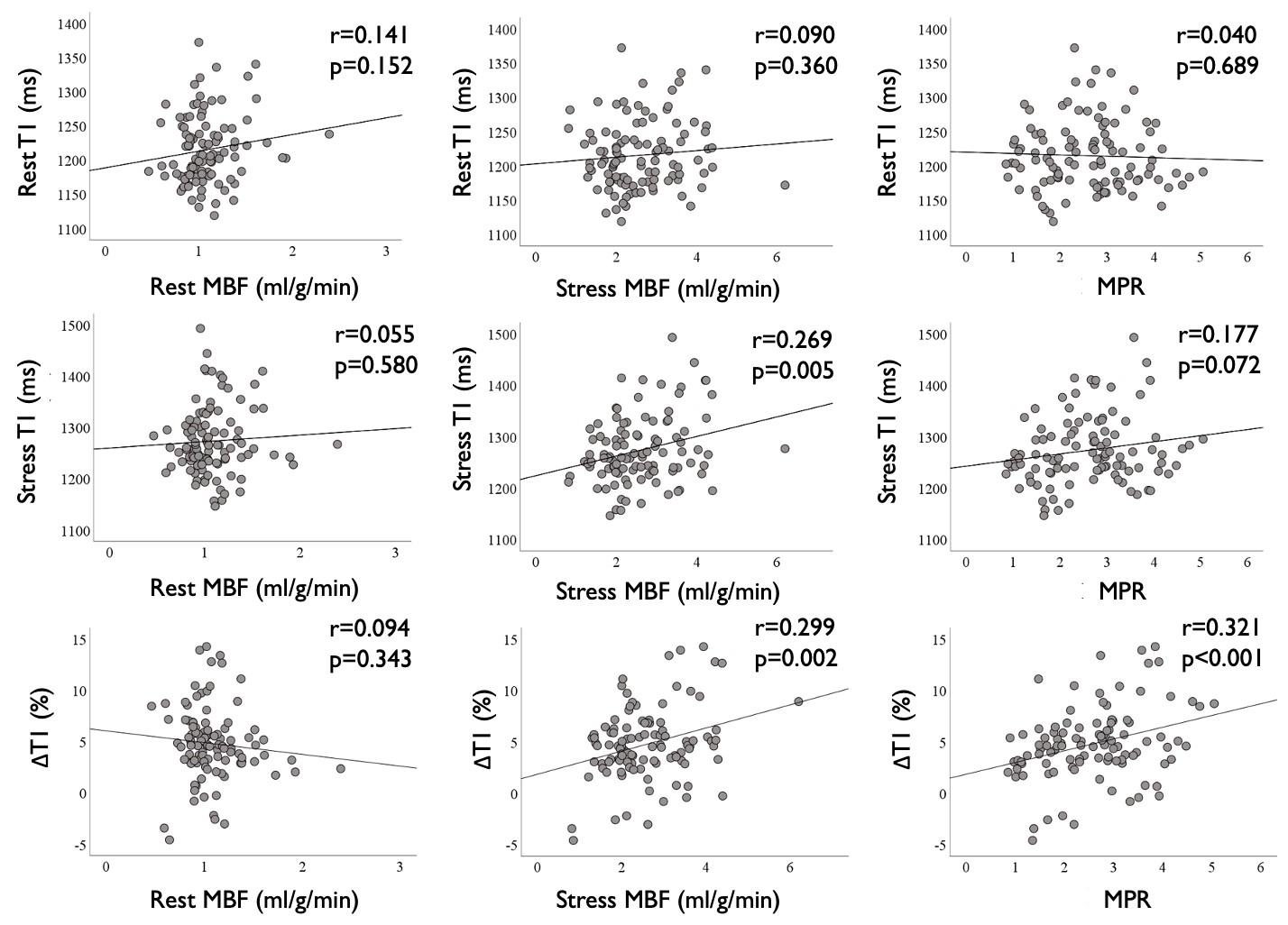

Supplement: jeaf059_Supplementary_Data [file jeaf059_supplementary_data.docx]
